# Performance Testing of PCR Assay in Blood Samples for the Diagnosis of Toxoplasmic Encephalitis in AIDS Patients from the French Departments of America and Genetic Diversity of *Toxoplasma gondii*: A Prospective and Multicentric Study

**DOI:** 10.1371/journal.pntd.0004790

**Published:** 2016-06-29

**Authors:** Daniel Ajzenberg, Isabelle Lamaury, Magalie Demar, Cyrille Vautrin, André Cabié, Stéphane Simon, Muriel Nicolas, Nicole Desbois-Nogard, Rachida Boukhari, Homayoun Riahi, Marie-Laure Dardé, Patrice Massip, Michel Dupon, Pierre-Marie Preux, Anaïs Labrunie, Marie-Paule Boncoeur

**Affiliations:** 1 INSERM, UMR_S 1094, Neuroépidémiologie Tropicale, Université de Limoges; Laboratoire de Parasitologie-Mycologie, Centre Hospitalier Universitaire de Limoges, Limoges, France; 2 Service de Maladies Infectieuses et Tropicales, Centre Hospitalier Universitaire de Pointe-à-Pitre, Pointe-à-Pitre, Guadeloupe, France; 3 Equipe EA3593—Ecosystemes Amazoniens et Pathologie Tropicale, Université de la Guyane, Guyane française, Cayenne, France; Laboratoire de Parasitologie-Mycologie, Centre hospitalier de Cayenne, Cayenne, Guyane française, France; 4 Service de Médecine, Centre Hospitalier de l'Ouest Guyanais, Saint-Laurent du Maroni, Guyane française, France; 5 INSERM CIC1425, Centre Hospitalier Universitaire de Martinique, Fort de France, Martinique, France; 6 Laboratoire de Microbiologie, Centre Hospitalier Universitaire de Pointe-à-Pitre, Pointe-à-Pitre, Guadeloupe, France; 7 Laboratoire de Parasitologie-Mycologie-Immunologie, Centre Hospitalier Universitaire de Martinique, Fort de France, Martinique, France; 8 Service de Biologie Médicale, Centre Hospitalier de l'Ouest Guyanais, Saint-Laurent du Maroni, Guyane française, France; 9 Laboratoire de Parasitologie-Mycologie, Centre Hospitalier Universitaire de Limoges, Limoges, France; 10 Service des Maladies Infectieuses et Tropicales, Hôpital Purpan, Centre hospitalier universitaire de Toulouse, Toulouse, France; 11 Service des Maladies Infectieuses et Tropicales, Hôpital Pellegrin, Centre hospitalier universitaire de Bordeaux, Bordeaux, France; 12 INSERM, UMR_S 1094, Neuroépidémiologie Tropicale, Université de Limoges; Centre d’Epidémiologie de Biostatistique et de Méthodologie de la Recherche (CEBIMER), Centre Hospitalier Universitaire de Limoges, Limoges, France; 13 INSERM, UMR_S 1094, Neuroépidémiologie Tropicale, Université de Limoges; Service de Neuroradiologie, Centre Hospitalier Universitaire de Limoges, Limoges, France; University of Minnesota, UNITED STATES

## Abstract

**Background:**

Toxoplasmic encephalitis in patients with AIDS is a life-threatening disease mostly due to reactivation of *Toxoplasma gondii* cysts in the brain. The main objective of this study was to evaluate the performance of real-time PCR assay in peripheral blood samples for the diagnosis of toxoplasmic encephalitis in AIDS patients in the French West Indies and Guiana.

**Methodology/Principal Findings:**

Adult patients with HIV and suspicion of toxoplasmic encephalitis with start of specific antitoxoplasmic therapy were included in this study during 40 months. The real-time PCR assay targeting the 529 bp repeat region of *T*. *gondii* was performed in two different centers for all blood samples. A Neighbor-Joining tree was reconstructed from microsatellite data to examine the relationships between strains from human cases of toxoplasmosis in South America and the Caribbean. A total of 44 cases were validated by a committee of experts, including 36 cases with toxoplasmic encephalitis. The specificity of the PCR assay in blood samples was 100% but the sensitivity was only 25% with moderate agreement between the two centers. Altered level of consciousness and being born in the French West Indies and Guiana were the only two variables that were associated with significantly decreased risk of false negative results with the PCR assay.

**Conclusion/Significance:**

Our results showed that PCR sensitivity in blood samples increased with severity of toxoplasmic encephalitis in AIDS patients. Geographic origin of patients was likely to influence PCR sensitivity but there was little evidence that it was caused by differences in *T*. *gondii* strains.

**Trial Registration:**

ClinicalTrials.gov NCT00803621

## Author Summary

Diagnosis of toxoplasmic encephalitis (TE) in patients with AIDS is not straightforward because clinicians rely initially on an empiric diagnosis based on clinical and radiographic improvement to specific anti-*Toxoplasma gondii* therapy. There is therefore a need for biological tools to improve the diagnosis of TE, especially in tropical areas where this diagnosis is likely to be underestimated. The use of PCR testing in blood samples for the diagnosis of TE has been limited by its poor sensitivity in the studies conducted in Europe. In tropical South America, the results of PCR sensitivity in blood samples were controversial. Considering that *T*. *gondii* strains from tropical South America have substantial genetic and pathogenic differences with those from USA and Europe, it is therefore important to re-evaluate the performance of the PCR assay in blood samples for the diagnosis of TE in AIDS patients from this region. Our results showed that the only interest of PCR would be in the most severe forms of TE with altered consciousness because PCR is more likely to be positive. We also provided important genotyping data on *T*. *gondii* strains isolated in human cases of toxoplasmosis in the Caribbean and in South America.

## Introduction

The protozoan *Toxoplasma gondii* is a cosmopolitan parasite that virtually infects all warm-blooded animals, including humans who become infected postnatally by ingesting tissue cysts from undercooked meat, consuming food contaminated with oocysts, or by accidentally ingesting oocysts from the environment [1]. The genetic diversity of this parasite is limited to a few successful clonal lineages in North America, Europe, Africa and China but is considerably higher in tropical South America [2]. In non-immunocompromised persons, toxoplasmosis is usually asymptomatic or limited to a mild symptomatology except in tropical South America. The prevalence of acquired and congenital ocular toxoplasmosis is much higher in Brazil and Colombia than in another place in the world and the Amazonian toxoplasmosis is a disseminated infection that requires management in intensive care units even in otherwise healthy adults [3, 4]. There is more and more evidence that the greater severity of toxoplasmosis in South America results from poor host adaptation to the genetically diverse *T*. *gondii* strains from this region [5].

Toxoplasmic encephalitis (TE) in patients with AIDS is a life-threatening disease mostly due to reactivation of *Toxoplasma gondii* cysts in the brain. TE can be inaugural of AIDS in patients who are not aware of their HIV seropositivity, but poor compliance with cotrimoxazole prophylaxis in patients with CD4 cell counts <200/μL is the major event leading to TE [6]. The incidence of TE in AIDS patients has greatly decreased since the introduction of HAART, but HIV-associated toxoplasmosis hospitalizations remain substantial, even in the United States [7, 8]. TE must be treated with specific anti-toxoplasmic therapy as soon as the diagnosis of TE is clinically and radiologically suspected [9]. Cerebral biopsy showing *T*. *gondii* tachyzoites is the only way to make a definite diagnosis of TE but is rarely undertaken in AIDS patients at baseline. The clinical and radiological response to specific therapy is still the gold standard for confirming *a posteriori* the diagnosis of TE in AIDS patients. This presumptive diagnosis has important limitations since up to 40% of AIDS patients with suspected TE and treated with specific therapy could not have in fact TE [10].

Laboratory investigations are considered not helpful in the diagnosis of TE. The majority of patients have positive IgG and negative IgM against *T*. *gondii*, simply indicating that they acquired toxoplasmosis in the past, mostly during childhood. However, the negative predictive value of a negative serologic testing for toxoplasmosis is high because it is estimated that < 3% of patients with AIDS have no demonstrable antibodies to *T*. *gondii* at the time of diagnosis of TE [11]. The diagnostic performance of PCR tests in various biological samples, mostly CSF and blood, was regularly assessed since the early years of the AIDS pandemic [12]. Blood samples are the only ones that can be easily obtained from the patients without invasive procedures. Although specificity was high, the use of PCR testing in blood samples for the diagnosis of TE has been limited by its poor sensitivity in the studies conducted in Europe [12–16].A few studies have been conducted in tropical South America and the results of sensitivity were highly controversial [17–20]. Considering that *T*. *gondii* strains from tropical South America have substantial genetic and pathogenic differences with those from USA and Europe, it is therefore important to re-evaluate the performance of the PCR assay in blood samples for the diagnosis of TE in AIDS patients from this region.

The main objective of this study was to evaluate the performance of real-time PCR assay in peripheral blood samples for the diagnosis of TE in AIDS patients from the French departments of America. The French departments of America (DFA) are French tropical overseas departments that include French Guiana in mainland South America and the French West Indies islands of Martinique and Guadeloupe in the Caribbean. The secondary objective was to collect and genotype *T*. *gondii* isolates from these patients.

## Methods

### Ethics statement

This study was approved by the French Ethics Committee “Comité de Protection des Personnes du Sud-Ouest et Outre-Mer 4” on April 4, 2008, with the reference number cpp08-006a. The Toxo-DFA study is registered in the ClinicalTrials.gov database (Identifier: NCT00803621). All patients included in the study were adult and provided written informed consent.

### Patient population

The Toxo-DFA study is an epidemiological, prospective, and multicentric study to validate the real-time PCR assay in peripheral blood samples for the diagnosis of TE in AIDS patients from a tropical area. It was conducted between September 16, 2008, and December 30, 2011, in four hospital centers of the French departments of America: two in French Guiana (Cayenne and Saint-Laurent du Maroni) and two in the French West Indies (Fort de France in Martinique and Pointe à Pitre in Guadeloupe).

Study participants had to meet all of the following criteria: age >18 years, informed written consent, positive serologic test for HIV, and clinical and radiological suspicion of TE with start of specific antitoxoplasmic therapy. Patients legally protected or uncovered by social insurance, or with a specific antitoxoplasmic therapy already initiated since 72h or more were excluded from the study.

The collection of data was done in an online secured case report form with CS-ONLINE from CAPTURE SYSTEM software. Patients were assessed clinically at baseline, between days 6–8, 15–21, and 42–56. Neuroradiographic scans by computed tomography (CT) or magnetic resonance imaging (MRI) were performed at baseline, between days 15–21, and 42–56.

### Diagnosis of TE

The gold standard for diagnosing TE was based on the clinical and radiological responses to specific antitoxoplasmic therapy after suspicion of cerebral toxoplasmosis. A validation committee of independent experts reviewed and classified the cases in 4 categories. TE was considered definite when there was a complete or significant clinical and radiological response to specific therapy (with not necessarily disappearance of radiological and clinical lesions), and no elements for an alternative diagnosis. TE was considered probable when radiological lesions were compatible but only partial improvement was observed with specific therapy (due to non-optimal treatment or incomplete follow-up), and no elements for an alternative diagnosis. Absence of TE was considered definite when there was no improvement or worsening of lesions with specific therapy or absence of *T*. *gondii* in cerebral biopsy samples and presence of elements for an alternative diagnosis. Absence of TE was considered probable when there was no response to specific therapy and no elements for an alternative diagnosis.

### Laboratory investigations

Two different centers were involved in the laboratory investigations: the Limoges center in metropolitan France and the Cayenne center in French Guiana.

#### Laboratory investigations in the Limoges center

Blood samples were collected in two 5-mL sterilized tubes with EDTA for each patient included in the study and sent to the Limoges laboratory for DNA extraction, PCR assay, mouse bioassay, and genotyping analysis. The DNA extraction for PCR assay was performed with the QIAamp DNA kit (QIAGEN, Courtaboeuf, France) on Buffy coat collected after centrifugation of the first blood sample for 10 min at 1,500 g. Mouse inoculation of the second blood sample was carried out if the first blood sample had tested positive with the PCR assay. Genotyping analysis was performed on strains isolated by mouse bioassay.

The real-time PCR assay targeted the 529 bp repeat region (*REP529*, GenBank accession no. AF146527) of *T*. *gondii* DNA [21] and the protocol was adapted from [22]. The nucleotide sequences of the primers were 5′-AGGCGAGGGTGAGGATGA-3′ (nucleotide position: 269–286) and 5′-TCGTCTCGTCTGGATCGCAT-3′ (nucleotide position: 402–383). The nucleotide sequence of the Taqman probe was 5′-6-FAM-CGACGAGAGTCGGAGAGGGAGAAGATGT-BHQ1-3′ (nucleotide position: 308–335). The TaqMan probe was labeled with a fluorescent dye (6-carboxyfluorescein, 6-FAM) at 5′ end and a dark quencher (Black Hole Quencher, BHQ1) at the 3′ end. The primers and the Taqman probe were designed and purchased at Eurofins MWG Operon (Ebersberg, Germany). Real-time PCR reactions were performed on a Rotor-Gene 6000 (Corbett Life Science) in a final volume of 20 μL with 1X LightCycler FastStart DNA Master Hybridization Probes kit (Roche diagnostics, Mannheim, Germany), 0.5 U of uracyl DNA N-glycosylase (Roche Diagnostics, Mannheim, Germany), 5 mmol/L of MgCl2, 0.5 μmol/L of each primer, 0.1 μmol/L of the Taqman probe, and 5 μL of template DNA. The cycling conditions were 2 minutes at 50°C (activation of the uracil-N-glycosylase), followed by 10 minutes at 95°C (inactivation of uracil-N-glycosylase and DNA denaturation) and 50 cycles consisting of 20 seconds at 95°C and 40 seconds at 60°C. The results were expressed in cycle threshold (C_t_) values and the parasite load/mL blood was calculated with a standard curve obtained by plotting the Ct values against each standard of known concentration parasite DNA. All DNA samples were tested in duplicate and each assay was considered positive if at least one test of the duplicate was positive. Each PCR run included a negative control without DNA and, to check the absence of PCR inhibitors, each sample was coamplified with an internal positive control consisting of DNA extracted from two tachyzoites obtained from mouse peritoneal fluids infected with the RH strain.

*T*. *gondii* strains isolated by mouse bioassay were genotyped using 15 microsatellite markers distributed on 10 of 14 chromosomes, as described previously [23]. For comparison, we included the genotyping data of three reference strains and of 43 strains collected from human cases of toxoplasmosis in the Caribbean and in South America by the French national reference center for toxoplasmosis (Table 1). The GT1, ME49, and VEG strains were used as reference *T*. *gondii* type I, II, and III strains, respectively. Type II and III strains are common in Europe, North America, Eastern Africa, and temperate South America whereas type I strains have been isolated from all continents but very infrequently [2]. Ten strains (BRA01–10) were collected in patients infected with imported strains whose epidemiological and genotyping investigation revealed that the origin of infection was likely Brazil. Ten strains were collected in patients infected in the French West Indies: GLP01–05 strains were from Guadeloupe and MTQ01–05 from Martinique. French Guiana consists of two different environments: a large wild environment characterized by the unpopulated Amazonian rainforest and a thin anthropized environment where people live along the Atlantic coast [24]. There is a clear genetic differentiation between *T*. *gondii* strains from the two environments. Wild strains are genetically highly divergent while strains from the anthropized environment are much less diverse and are similar to other strains seen in the Caribbean [25]. The clinical features of toxoplasmosis in the two environments are also very different. Consumption of wild game or drinking untreated water from the rainforest is at risk of being infected with wild strains that are associated to the most severe form of toxoplasmosis ever described in otherwise healthy adults, the Amazonian toxoplasmosis [4]. Such cases are rare and the majority of the population who lives in the anthropized area is infected with strains that are associated with classical symptoms of toxoplasmosis. For these reasons, we separated the strains from French Guiana in two categories: Strains AMZ 1–18 were collected in patients with Amazonian toxoplasmosis who had been infected with wild strains from the rainforest, whereas strains GUF01–05 were from toxoplasmosis cases diagnosed in babies with congenital toxoplasmosis or immunocompromised patients living in the anthropized areas of French Guiana. All strains were also designated with a code according to the nomenclature for *T*. *gondii* isolates of the French national reference center for toxoplasmosis and the *Toxoplasma* Biological Resource Center (BRC) (http://www.toxocrb.com) (Table 1).

**Table 1:** Genotyping analysis with 15 microsatellite markers of 3 reference type I, II, and III strains, the HTI01 strain isolated in the present study, and 43 strains collected in human cases of toxoplasmosis from South America and the Caribbean region by the French national reference center for toxoplasmosis.

				Microsatellite markers														
Isolate	Year	BRC^a^ code	Data	*TUB2*	*W35*	*TgM-A*	*B18*	*B17*	*M33*	*MIV*.*1*	*MXI*.*1*	*M48*	*M102*	*N60*	*N82*	*AA*	*N61*	*N83*
GT1	1980	TgA00004	REF I	291	248	209	160	342	169	274	358	209	168	145	119	265	087	306
ME49	1965	TgA00001	REF II	289	242	207	158	336	169	274	356	215	174	142	111	265	091	310
VEG	1988	TgH00005	REF III	289	242	205	160	336	165	278	356	213	188	153	111	267	089	312
HTI01	2009	TgH40001	TI^b^	289	242	207	162	336	169	274	356	237	166	142	111	281	093	312
AMZ01	2003	TgH18005	AMZT^c^	289	242	203	160	344	165	276	356	211	168	142	109	271	087	320
AMZ02	2002	TgH18002	AMZT	289	246	203	160	337	165	274	356	209	172	136	111	251	109	310
AMZ03	2001	TgH18001	AMZT	289	246	203	160	344	167	272	356	229	176	142	113	263	085	312
AMZ04	2004	TgH18009	AMZT	291	242	203	160	338	167	274	356	209	188	138	115	263	091	312
AMZ05	2002	TgH18003	AMZT	291	242	203	160	339	165	272	358	221	174	138	107	277	095	312
AMZ06	2004	TgH18013	AMZT	291	242	203	160	346	167	272	356	217	170	147	127	257	085	310
AMZ07	1997	TgH00009	AMZT	291	242	203	162	344	167	276	356	217	170	142	113	277	091	308
AMZ08	2011	TgH18051	AMZT	291	242	205	160	340	165	276	356	211	176	138	105	269	087	316
AMZ09	2010	TgH42001	AMZT	291	246	203	158	346	169	272	356	229	172	142	105	261	105	316
AMZ10	2007	TgH18028	AMZT	291	246	203	160	326	167	276	356	211	178	147	111	259	087	306
AMZ11	2009	TgH18039	AMZT	291	246	203	160	342	171	274	356	213	172	149	105	273	089	318
AMZ12	2012	TgH19006	AMZT	293	246	203	162	344	171	272	356	211	182	138	105	267	091	302
AMZ13	2012	TgH19008	AMZT	291	246	203	160	334	167	274	356	209	174	142	109	201	085	306
AMZ14	2003	TgH18004	AMZT	291	246	205	166	338	165	274	356	213	172	138	119	259	083	312
AMZ15	2011	TgH18048	AMZT	293	242	203	162	344	167	274	358	217	176	138	115	273	085	316
AMZ16	2003	TgH18007	AMZT	289	242	203	158	344	171	272	356	209	182	149	121	265	089	317
AMZ17	2004	TgH18008	AMZT	289	246	203	158	338	167	276	354	213	168	138	111	281	093	318
AMZ18	2006	TgH18021	AMZT	291	246	205	166	334	167	272	356	213	174	151	107	267	087	325
BRA01	2009	TgH23028	CT^d^	289	242	205	160	342	165	278	358	233	164	145	111	316	089	308
BRA02	2000	TgH24001	CT	289	242	205	160	342	165	278	358	237	164	145	111	316	089	308
BRA03	2012	TgH14011	CT	289	242	205	162	334	169	272	356	227	164	142	117	334	087	308
BRA04	2014	TgH34032	TI	289	242	205	162	344	165	278	358	223	164	142	111	263	105	312
BRA05	2005	TgH25010	CT	289	242	205	162	344	165	278	358	225	164	142	111	263	111	312
BRA06	2009	TgH23018	TI	289	242	205	162	344	165	278	358	225	164	142	111	269	089	312
BRA07	2011	TgH13117	TI	289	242	205	162	344	165	278	358	227	164	142	111	263	119	312
BRA08	1994	TgH32005	CT	291	242	205	160	362	165	278	356	225	174	140	111	265	091	314
BRA09	2006	TgH32069	CT	291	242	205	162	362	165	278	356	223	166	151	111	263	093	337
BRA10	1991	TgH00006	CT	291	242	205	162	362	169	272	358	221	166	142	111	332	095	339
GLP01	2010	TgH40002	CT	291	242	205	162	336	165	278	356	211	164	147	109	277	087	312
GLP02	1992	TONT	CT	289	242	205	162	336	165	278	356	213	164	142	111	279	087	312
GLP03	2009	TgH29086	TI	291	242	205	162	342	165	278	356	211	164	142	109	277	087	312
GLP04	2002	TgH29005	TI	291	242	205	162	342	165	278	356	211	164	142	109	277	087	312
GLP05	2005	TgH13006	TI	291	242	205	162	342	165	278	356	213	164	142	109	277	087	312
GUF01	2002	TgH20001	CT	291	242	205	162	336	165	278	356	213	164	142	109	267	089	312
GUF02	2004	TgH13013	TI	289	242	205	162	336	165	278	356	229	164	147	111	261	089	312
GUF03	2014	TgH18057	CT	291	248	209	160	342	165	278	354	209	166	140	113	277	095	304
GUF04	2014	TgH19010	CT	289	242	205	160	336	165	278	356	209	190	147	111	265	089	312
GUF05	2007	TgH18022	NR^e^	289	248	209	160	336	165	278	356	209	166	149	121	271	087	306
MTQ01	2007	TgH16001	TI	289	242	207	158	336	169	274	356	213	174	147	119	271	089	335
MTQ02	2007	TgH16003	TI	291	242	205	162	342	165	278	356	211	164	142	109	277	087	312
MTQ03	2010	TgH16004	CT	289	244	207	158	336	169	274	356	211	176	140	113	259	101	310
MTQ04	2013	TgH16008	CT	291	242	205	162	336	165	278	356	213	164	142	111	277	087	312
MTQ05	2013	TgH16010	CT	291	242	205	162	336	165	278	356	213	164	142	111	283	085	312

^a^BRC, *Toxoplasma* Biological Resource Center.

^b^TI, toxoplasmosis in patient with immunosuppression.

^c^AMZT, Amazonian toxoplasmosis.

^d^CT, congenital toxoplasmosis.

^e^NR, not reported.

#### Laboratory investigations in the Cayenne center

At the end of the study, all DNA samples that had been tested with the PCR assay in Limoges and stored at -80°C were sent in Cayenne, French Guiana, for being retested with the PCR assay developed in this laboratory. *T*. *gondii*-specific primers and the TaqMan probe were selected from the 529 bp repeat region (*REP529*, GenBank accession no. AF146527) and adapted from [21]. The primers and probe were designed using Primer Express Software version 3.0 (Applied Biosystem by Life Technologies Corp., Carlsbad, CA). Primer sequences were 5′-GCTCCTCCAGCCGTCTTG-3′ (nucleotide position: 224–241) and 5′-TCCTCACCCTCGCCTTCAT-3′ (nucleotide position: 283–265), and the TaqMan probe was 5′-6-FAM-AGGAGAGATATCAGGACTGTA-NFQ-MGB-3’ (nucleotide position: 243–263). The TaqMan probe was labeled with a fluorescent dye (6-carboxyfluorescein, 6-FAM) at 5′ end and a non-fluorescent quencher coupled with a minor groove binder (NFQ-MGB) at the 3′ end. Real-time PCR reactions were carried out in final volumes of 25 μL with 1X TaqMan Gene Expression Master Mix containing uracyl DNA N-glycosylase (Applied Biosystems), 0.9 μmol/L of each primer, 0.25 μmol/L of the probe, and 5 μL of template DNA. All reactions were run on the 7300 Real-Time PCR System (Applied Biosystems) and the cycling conditions were 2 minutes at 50°C (activation of the uracil-*N*-glycosylase), followed by 10 minutes at 95°C (inactivation of uracil-N-glycosylase and DNA denaturation) and 40 cycles consisting of 15 seconds at 95°C and 1 minute at 60°C. The results were expressed in cycle threshold (C_t_) values and the parasite load/mL blood was calculated with a standard curve obtained by plotting the Ct values against each standard of known concentration parasite DNA. All DNA samples were tested in duplicate and each assay was considered positive if at least one test of the duplicate was positive. Each PCR run included a negative control without DNA and the TaqMan Exogenous Internal Positive Control (IPC, Applied Biosystems) was used to check the absence of PCR inhibitors in each sample.

### Neighbor-joining clustering and statistical analysis

A Neighbor-Joining tree was reconstructed from microsatellite data to examine the relationships between strains collected from human cases of toxoplasmosis in South America and the Caribbean. The tree was constructed with Populations 1.2.32 (http://bioinformatics.org/populations/) based on Cavalli-Sforza and Edwards chord-distance estimator [26] and generated with MEGA 6.05 (http://www.megasoftware.net/history.php) software.

The software used for statistical analyses was SAS 9.3 and the significant threshold of the p value was 0.05 (SAS Institute, Cary, USA). Median and interquartile intervals were given for quantitative variables while qualitative variables were presented as sample size and percentages. Nonparametric tests were used for comparing variables between patients with TE and those without TE: a Fisher exact test was performed for the qualitative variables and a Mann-Whitney test was used for the quantitative variables. To evaluate the diagnostic performance of the PCR assay in detecting TE in peripheral blood samples from AIDS patients in the French West Indies and Guiana, the reliability and the validity of the test were assessed. For these analyses, cases reviewed by the validation committee with definite or probable TE were coded positive and those with definite or probable absence of TE were coded negative. The reliability of the PCR assay was estimated by the Cohen’s Kappa coefficient of agreement and its 95% confidence interval between results of the test in Cayenne and Limoges centers. The validity of the PCR assay was estimated by the sensitivity and the specificity of the test in detecting definite and probable cases of TE that had been identified by the validation committee. The 95% confidence intervals of sensitivity and specificity were estimated by the exact method. A study of the false negative results was carried out to search explanatory factors according to the sample size of this sub-group by using a logistic regression model with the false negative status as the response variable and the potential associated factors as the explicative variables.

## Results

### Patient characteristics and diagnosis of TE

A total of 46 patients were included in this study: 17 in French Guiana (8 in Cayenne and 9 in Saint-Laurent du Maroni) and 29 in the French West Indies (23 in Guadeloupe and 6 in Martinique).

The validation committee reviewed the cases as follows: 2 cases were not assessable because of insufficient data (both included in the center of Saint Laurent du Maroni in French Guiana), 36 cases were classified in the TE group and 8 in the non-TE group. In the TE group, 30 were classified as definite TE and 6 as probable TE. In the non-TE group, 6 were classified as definite absence of TE and 2 as probable absence of TE. The demographic, laboratory and clinical baseline characteristics of the 44 patients classified in TE and non-TE groups by the validation committee are available in Table 2. There was no statistical difference between patients with TE and those without TE with respect to the variables listed in Table 2 except for the place of birth and the results of *T*. *gondii* serology.

**Table 2:** Demographic, laboratory and clinical baseline characteristics of all patients, of patients with a final diagnosis of TE, and of patients without a final diagnosis of TE, French West Indies and Guiana, 2008–2011.

Variable	All patients	Patients with TE^a^	Patients without TE	p value
Demographical data	n = 44	n = 36	n = 8	
Male sex	28 (63.64)	22 (61.11)	6 (75.00)	0.69
Age, median years (IQR^b^)	43.00 (36.50–50.0)	42.50 (35.00–48.00)	45.50 (38.50–62.50)	0.29
Born in the French West Indies & Guiana	29 (65.91)	21 (58.33)	8 (100.00)	0.04
Born in the Caribbean	32 (72.73)	26 (71.29)	6 (75.00)	1.00
*T*. *gondii* serology	n = 41	n = 33	n = 8	
IgG positive and IgM negative	39 (95.12)	33 (100.00)	6 (75)	0.03
CD4^+^ cell count	n = 37	n = 29	n = 8	
Median cells/μL (IQR)	43.00 (21.00–128.00)	43.00 (15.00–122.00)	58.50 (27.50–137.00)	0.74
Range	3–465	3–243	5–465	
Clinical data	n = 44	n = 36	n = 8	
No prophylaxis against TE	36 (81.82)	29 (80.56)	7 (87.50)	1
Fever	11 (25.00)	9 (25.00)	2 (25.00)	1.00
Headache	18 (40.91)	17 (47.22)	1 (12.50)	0.11
Motor-sensory defects	22 (50.00)	19 (52.78)	3 (37.50)	0.70
Seizures	9 (20.45)	8 (22.22)	1 (12.50)	1.00
Altered level of consciousness	10 (22.73)	8 (22.22)	2 (25.00)	1.00
Diffuse neuropsychic signs	19 (43.18)	17 (47.22)	2 (25.00)	0.43

Data are no. (%) of patients, unless otherwise indicated.

^a^TE, Toxoplasmic encephalitis.

^b^IQR, interquartile range.

Two different classifications were used for clustering the 44 patients into two groups according to their place of birth. In the first classification, the first group gathered the 29 patients who were born in the French West Indies and Guiana (6 in French Guiana, 17 in Guadeloupe, and 6 in Martinique) while the 15 patients who were born elsewhere were put together in a second group (7 in Haiti, 3 in Brazil, 2 in Suriname, 1 in Dominica, 1 in Dominican Republic, and 1 in Spain). Being born in the French West Indies and Guiana was significantly more common in patients without TE than in those with TE (p = 0.04) because all patients without TE (n = 8) were born in the French West Indies and Guiana (Table 2). The second classification was based on geography with 32 patients born in the Caribbean (17 in Guadeloupe, 7 in Haiti, 6 in Martinique, 1 in Dominica, and 1 in Dominican Republic), 11 in South America (6 in French Guiana, 3 in Brazil, 2 in Suriname), and 1 in Europe. According to this second classification, being born in the Caribbean was not statistically different between patients with TE and those without TE (Table 2).

Of the 41 patients with available data on *T*. *gondii* serology, only 2 had negative test results for IgG and IgM against *T*. *gondii*, and both of them had definite absence of TE (Table 2). The blood samples of the remaining 39 patients tested positive for IgG and negative for IgM, indicating past immunization against *T*. *gondii*. At presentation, only 7 patients were receiving systemic antiprotozoal prophylaxis: trimethoprim-sulfamethoxazole (cotrimoxazole, n = 5), pyrimethamine (n = 1), and atovaquone (n = 1). Of the 5 patients with cotrimoxazole prophylaxis, good compliance was reported in only one patient.

The choice of specific empirical antitoxoplasmic first-line therapy was based on routine practice of each center: all patients from French Guiana (n = 15), five out of six patients from Martinique and only one patient from Guadeloupe were treated with trimethoprim-sulfamethoxazole (cotrimoxazole) whereas 22 out of 23 patients from Guadeloupe were given a pyrimethamine-based combination with either sulphadiazine (n = 16) or clindamycin (n = 6). One patient was treated with a combination of pyrimethamine plus atovaquone in Martinique. Five (11.4%) patients, all with TE, died within the first 12 weeks after antitoxoplasmic therapy was begun.

### Performance of the PCR assay

Of the 44 patients, the PCR assay tested positive in blood samples of 9 patients with TE and tested negative in 35 patients (27 in the TE group and 8 in the non-TE group). The sensitivity was 25.00% (95% CI 12.12–42.20) which is low, and the specificity was 100% (95%CI 63.06–100.00) which is maximal. Of the 9 blood samples with a positive PCR test, only 4 were detected simultaneously in both Limoges (Parasites/mL blood: 0.01–0.13, 0–0.42, 4.67–6.49, 2.30–8.80) and Cayenne (Parasites/mL blood: 1.46–1.83, 0.45–0.53, 15.38–22.25, 2.15–9.83, respectively) laboratories, whereas 4 were detected in the Cayenne laboratory alone (Parasites/mL blood: 0.02–0.24, 0.01–0.18, 0.37–0.98, 4.80–8.30) and 1 in the Limoges laboratory alone (Parasites/mL blood: DNA: 0–1.16). The sensitivity was 11.11% (95% CI 3.11–26.06), 13.89% (95% CI 4.67–29.50), and 22.22% (95% CI 10.12–39.15) when positive PCR tests were observed in both centers, in the Limoges center, and in the Cayenne Center, respectively. The Cohen's kappa coefficient used to estimate the agreement between results of PCR tests performed in both centers was 0.5528 (95% CI 0.2112–0.8945), which indicates moderate agreement [27]. From these data, we can conclude that the majority of PCR results were close to the limits of detection and the difference in detection of *T*. *gondii* DNA at the two centers were due to differences in the analytical sensitivity of the PCR assay at each site.

### Factors associated with false negative results with the PCR assay in blood samples

The relationship between the risk of having a false negative result with the PCR assay in the blood for the diagnosis of TE and different baseline variables is shown in Table 3. Altered level of consciousness and being born in the French West Indies and Guiana were the only two variables that were associated with significantly decreased risk of false negative results with the PCR assay according to multivariate logistic regression analysis.

**Table 3:** Univariate or multivariate logistic regression analysis of baseline characteristics that significantly correlated with a false negative result of the PCR assay in the blood for the diagnosis of toxoplasmic encephalitis in patients with AIDS, French West Indies and Guiana, 2008–2011.

	Univariate analysis		Multivariate analysis^b^	
Variable^a^	OR (95% CI)	p value	OR (95% CI)	p value
CD4 cell count <25 cells/μL	0.54 (0.11–2.53)	0.43	-	
Fever	1.14 (0.28–4.67)	0.86	-	
Headache	2.23 (0.61–8.07)	0.22	-	
Motor-sensory defects	0.82 (0.24–2.78)	0.76	-	
Seizures	2.62 (0.48–14.49)	0.27	-	
Altered level of consciousness	0.18 (0.04–0.83)	0.03	0.07 (0.01–0.67)	0.02
Born in the French West Indies & Guiana	0.14 (0.03–0.75)	0.02	0.06 (0.01–0.61)	0.02
Born in the Caribbean	1.19 (0.31–4.60)	0.80	-	

^a^The sample size was 44 patients except for the variable CD4 cell count <25 cells/μL (n = 37).

^b^Multivariate analysis was performed only on variables with p value <0.2 for association with a false negative result of the PCR assay by univariate analysis.

### *T*. *gondii* strain isolation and genotyping analysis

**Fig 1:**
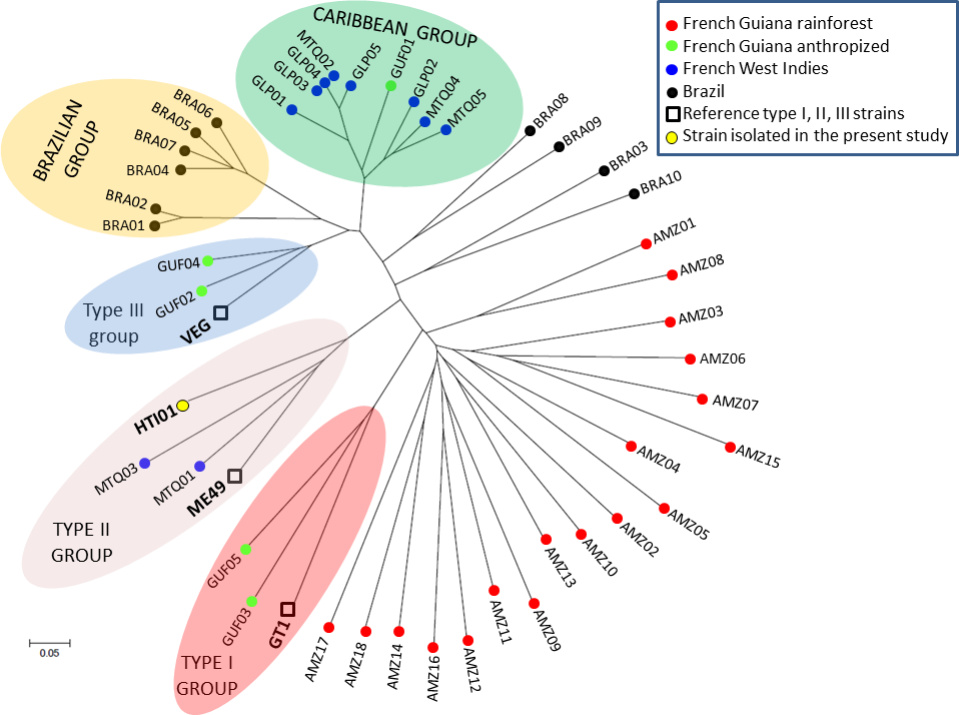
Neighbor-joining clustering of *T*. *gondii* strains based on 15 microsatellite markers. Squares are for the type I (GT1), II (ME49), and III (VEG) reference strains; the yellow point is for the HTI01 strain isolated in the present study; black points (BRA01–10) are for strains collected in patients infected with imported strains whose epidemiological and genotyping investigation revealed that the origin of infection was likely Brazil; red points (AMZ 1–18) are for strains collected in patients with Amazonian toxoplasmosis who had been infected with wild strains from the rainforest of French Guiana; green points (GUF01–05) are for strains from toxoplasmosis cases diagnosed in babies with congenital toxoplasmosis or immunocompromised patients living in the anthropized areas of French Guiana; blue points are for strains collected in patients infected in Guadeloupe (GLP01–05) and Martinique (MTQ01–05), both in the French West Indies.

Five blood samples that tested positive with the PCR assay in the Limoges laboratory were inoculated into mice and only one strain was isolated. This strain was isolated from a patient who was living in Guadeloupe but born in Haiti. The blood sample of this patient tested positive with the PCR assay in both laboratories of Limoges and Cayenne, and corresponded to the sample with the highest parasite load (Parasites/mL blood: 4.67–6.49 and 15.38–22.25, respectively). This strain was designated HTI01 in this study and cryopreserved at the *Toxoplasma* BRC with the denomination code TgH40001. The genotype of this strain with 15 microsatellite markers was compared with the genotypes of the three reference type I, II, and III strains and with those of 43 strains collected in human cases of toxoplasmosis from South America and the Caribbean region (Table 1). The neighbor-joining analysis clustered the HTI01 strain collected in the present study with the type II reference strain in the unrooted tree (Fig 1). The 43 strains collected from human cases of toxoplasmosis in the Caribbean and in South America by the French national reference center for toxoplasmosis were clustered as follows: i) strains collected in patients from the French West Indies were highly structured in only two groups: 2 strains clustered with the HTI01 strain in the type II group and 8 strains were grouped into a separate cluster called Caribbean group; ii) the anthropized strains from French Guiana were also highly structured into three groups: 2 strains were assembled in the type I group, 2 in the type III group, and 1 in the Caribbean group; iii) the 18 wild strains from the Amazonian forest of French Guiana were found on separate long branches and were highly divergent from all the other strains, as reported previously [24, 25]; iv) of the 10 Brazilian strains, six were structured in one separate group called Brazilian group and four strains were divergent and found on separate long branches like wild strains from French Guiana.

## Discussion

There is a need of treatments and diagnostic tools for TE adapted to AIDS patients in the specific context of tropical areas. For example, the standard therapy of TE is the combination of pyrimethamine and sulfadiazine [9, 28]. However this treatment has several limitations, such as its cost, the high frequency of adverse reactions in AIDS patients, the absence of intravenous formulation and its frequent unavailability in poor resource settings. For these reasons, cotrimoxazole is often preferred as a first line therapy of TE in AIDS patients in tropical areas because it is efficacious, cheap, better tolerated, with an intravenous formulation and, most of all, is widely available in developing countries [29, 30]. Diagnosis of TE is not straightforward because the majority of clinicians rely initially on an empiric diagnosis based on clinical and radiographic improvement to specific anti-*T*. *gondii* therapy in the absence of a likely alternative diagnosis [28]. In tropical areas, many patients are diagnosed with HIV only after developing opportunistic infections such as TE and the differential diagnosis of focal neurological disease in patients with AIDS can be complex in the context of poor-resource settings [29]. Under-diagnosis is likely to be the consequence of the difficulties with diagnosing TE in tropical areas [31]. In this study, we aimed at evaluating the diagnostic performance of TE with the real-time PCR assay in peripheral blood samples from AIDS patients in a tropical setting. A total of 44 patients, 36 with TE and 8 without TE, from the French West Indies and Guiana in the French departments of America were included in the present study. The standards of healthcare are close to those of mainland France but this region is a crossroads for poor Caribbean and South American people who emigrate there for socio-economic reasons [32]. In this region, the HIV epidemic is a major public health problem and TE is a leading cause of death among HIV-infected adults [33, 34].

All patients without TE tested negative with the PCR assay in blood samples and all patients with a positive PCR result had TE. The 100% specificity of the PCR assay in blood samples for the diagnosis of TE in patients with AIDS in our study confirms the very high specificity of this test reported in the literature (median 99%, IQR 93.1%–100%) [12–18, 20]. However, with a sensitivity of 25%, the capacity of the PCR assay to detect TE in blood samples from patients with AIDS is low in a tropical area like the French departments of America. In fact, the sensitivity of this test to diagnose TE in blood samples from patients with AIDS seems to vary with geography in the literature. According to 5 studies conducted in the 1990s [12–16], the sensitivity ranged from 13.3% to 29.5% in Europe (median 24.3%, IQR 17.9–25.6) which is similar to the result of our study although this latter was conducted in a tropical area. In contrast, 3 studies from tropical South America in the 2000s showed contradictory results with a sensitivity of 1.2% in north-east Brazil, 18.8% in Colombia, and 80% in south-east Brazil [17, 18, 20]. It is difficult to compare these studies from Europe and South America with our study in the French departments of America because the volumes of blood samples (1, 5, or 10 mL), DNA extraction protocols (buffy coat versus whole blood), DNA targets (*REP529*, *B1*, *TgRE1*, and *rDNA repetitive gene*), and even primers for the same DNA target (*B1* or *REP529*) were different. The 6 oldest studies performed a conventional PCR [12–17] whereas real-time PCR was done in the present work and in the most recent studies [18, 20]. The Brazilian study with the highest PCR sensitivity (80%) used a volume of 10 mL of blood sample and a conventional PCR assay targeting the *B1* gene with primers B22 and B23 [17].

The DNA extraction step is essential for detecting *T*. *gondii* in blood samples. A recent study in the animal model showed that it was preferable to use buffy coat rather than whole blood, but stressed the importance of the volume of blood sample to increase the sensitivity of PCR assay [35]. The volume of blood sample in most studies that evaluated the performance of the PCR assay in AIDS patients with TE was 5 or 10 mL [12–15, 17] except in 2 studies that used only 1 mL [18, 20]. Using a limited volume of blood sample might explain, in part, the poor sensitivity of the PCR assay reported in one Brazilian study [19]. The choice of the DNA target for the PCR assay is also important because studies that tested different targets showed different sensitivity results according to the target [14, 15]. Experts generally recommend the use of *REP529* DNA target and real-time PCR for reaching the highest sensitivity but they stress the importance of the proficiency of the laboratory performing the diagnosis and the need for optimization of PCR conditions [36]. It is therefore better to use a well-optimized PCR assay targeting the B1 gene with a conventional PCR assay in a reference laboratory rather than a non-optimized PCR assay targeting *REP529* with a real-time PCR assay in an inexperienced laboratory [37]. The use of different primers for the same target may also lead to different results of sensitivity, as suggested in one study [15]. In our study, the target was *REP529* in Limoges and Cayenne laboratories but, because each center independently developed their own laboratory-optimized PCR assay for routine diagnosis of toxoplasmosis, primers were different and the sensitivity in each center was also different (13.89% and 22.22%, respectively). However, it is also true that identical primers can give variable results of sensitivity depending on the laboratory, which underlines, once again, the crucial importance of PCR optimization [36]. What makes consensus is the systematic use of uracyl DNA N-glycosylase (UDG) to avoid false-positive results caused by carry-over contaminations and an internal positive control (IPC) to avoid false-negative results caused by PCR inhibitors of PCR [38]. Such basic precautions were taken in the present study, in 3 studies from Europe, and in 1 study from Colombia [12, 14, 15, 20]. One study in Europe and one in Brazil reported the use of IPC but not UDG with sensitivities of 20% and 1.2%, respectively [13, 18]. The two studies that performed PCR without IPC and UDG reported a sensitivity of 25% in Europe and 80% in Brazil [16, 17].

Altogether, it seems that the methodological issues raised here cannot entirely explain the huge difference between sensitivities of the PCR assay in blood samples for diagnosing TE in AIDS patients from 4 tropical areas: 1.2% in patients from Recife, north-east Brazil [18], 18.8% in Colombia [20], 25% in the French West Indies and Guiana (this study), and 80% in São Paulo, south-east Brazil [17]. In the present study, the geographic origin of patients was likely to influence the sensitivity of the PCR assay because being born in the French West Indies and Guiana was a variable significantly associated with a decreased risk of false negative results according to multivariate logistic regression analysis. The first hypothesis to explain the link between geography and sensitivity of *T*. *gondii* DNA detection in blood samples is the strain hypothesis because the hotspot of *T*. *gondii* genetic diversity is in tropical South America and because the genotype of *T*. *gondii* strains is strongly linked to the presumed geographical origin of infection in immunocompromised patients [2, 39]. Most cases of TE result from local reactivation of brain cysts without parasitemia which explains the absence of detection of *T*. *gondii* in most blood samples. If some strains are more likely to disseminate in blood flow than others, this would have a strong effect on sensitivity of PCR in blood samples. For example, the sensitivity of PCR in blood samples is very low for diagnosing ocular toxoplasmosis in immunocompetent patients from France and *T*. *gondii* DNA is detectable only in ocular fluid samples [40, 41]. In contrast, *T*. *gondii* genotypes involved in ocular toxoplasmosis in south and south-east Brazil were not characterized from ocular fluid samples but from peripheral blood, and this prolonged parasitemia was confirmed by direct microscopic observation of tachyzoites in some blood samples [42–44]. Brazil is a big country with a complex *T*. *gondii* population structure and it is possible that such differences also exist at a regional scale in Brazil. If strains from south and south-east Brazil are more likely to disseminate in blood flow than those from the north-east, this could explain the regional variation of PCR sensitivity in blood samples for diagnosing TE in AIDS patients from Brazil [17, 18]. Another example of disseminating disease is the Amazonian toxoplasmosis whose diagnosis is always confirmed by a positive result of the PCR assay in blood samples despite the fact that the patients are not immunocompromised [4].

Little is known about the genetic background that characterizes disseminating strains but, based on what is known from wild strains of the Amazonian rainforest, the genotypes of these atypical strains are found on separate long branches in neighbor-joining trees and are highly divergent from the genotypes of all other strains, especially from the clonal type II and III strains that are common in Europe [24]. However, in the present study, we found little evidence that the effect of geographic origins on PCR sensitivity in blood samples for the diagnosis of TE in AIDS patients was caused by differences in *T*. *gondii* strains. Unfortunately, we isolated only one *T*. *gondii* strain in a patient who was not born in the French departments of America but in Haiti. The genotype of this strain was not atypical but rather related to type II which represents >95% of strains in Europe where the sensitivity of PCR assay is low in blood samples. The other patient who was not born in the French West Indies and Guiana and who had a positive PCR result was born in Spain and therefore also likely infected by a type II strain. If the strain hypothesis were true in our study, we would have expected positive PCR results in blood samples of the 3 patients from Brazil but none of them tested positive. In fact, the proportion of positive PCR results was higher in patients born in the French West Indies and Guiana (7/29, 24%) than in those born elsewhere (2/15, 13%).

We included in the analysis the genotyping data of 43 *T*. *gondii* strains collected by the French national reference center from patients infected in tropical South America and the Caribbean. Strains that infect humans in the French West Indies and anthropized areas of French Guiana were not found on separate long branches in the neighbor-joining tree like wild strains from the Amazonian rainforest or some strains from Brazil but were highly structured like in Europe. Type II and III strains that are common in Europe are also common in the French departments of America. The difference with Europe is the predominance of an endemic lineage called Caribbean group that comprises the Caribbean 1, 2 and 3 genotypes already described in domestic animals from the anthropized area of French Guiana and in immunocompromised patients from the French West Indies [24, 25, 39]. Although we did not isolate strains in patients born in the French departments of America in this study, it is likely that they were also infected by *T*. *gondii* strains belonging either to the types II and III lineages or to the Caribbean group but not to highly divergent strains that could have explained the better detection of *T*. *gondii* in blood samples for these patients. In the absence of a clear explanation by differences in *T*. *gondii* strains, the effect of geography on the sensitivity of PCR in blood samples of AIDS patients remains to be elucidated.

The main result of our study is that the sensitivity of PCR in blood samples increases with the severity of TE. The main severity factors of TE in AIDS patients are profound immunodepression and impaired consciousness. In a study conducted in AIDS patients with TE at admission in intensive care units, the factors independently associated with a poor outcome were a Glasgow coma scale ≤8 and a CD4 cell count <25/μL [45]. In our study, a CD4 cell count <25/μL was not associated with a decreased risk of false negative results with the PCR assay. However, altered level of consciousness was the second variable significantly associated with a decreased risk of false negative results according to multivariate logistic regression analysis. Of the 8 patients with altered level of consciousness and TE in our study, 5 (62.5%) tested positive in blood samples. All patients (n = 3) with a Glasgow coma scale ≤9 had a positive test result with the PCR assay in blood samples. The high PCR sensitivity of 80% in blood samples of the 64 patients from São Paulo, Brazil, could be explained by a high number of severe TE cases in this study but clinical data were not available [17].

In conclusion, the PCR assay in blood samples is not recommended for diagnosing TE in the tropical setting of the French departments of America areas because of a poor sensitivity. The only interest of PCR would be in the most severe forms of TE with altered consciousness because PCR is more likely to be positive. Even in these cases, it seems difficult to reach a good sensitivity with the PCR assay because the concentration of *T*. *gondii* DNA is very low. PCR protocols have to be perfectly optimized because positive PCR results rely on high Ct values that are at the limit of the detection of the method which jeopardizes a good agreement between diagnostic laboratories, as showed in our study. There is no argument that the PCR sensitivity could be influenced by the genetic background of *T*. *gondii* strains in this area even if the geographic origin of patients is likely to play a role for unclear reasons. We believe that our results can be expanded in any tropical setting with the exception of other parts of tropical South America, especially Brazil where *T*. *gondii* strain diversity is far more complex than in the French West Indies and the anthropized areas of French Guiana. Other studies are needed in Brazil to know whether genetic-based differences in the capacity of hematogenous dissemination of locally acquired *T*. *gondii* strains are likely to explain the considerable regional variations of the sensitivity of the PCR assay in blood samples of AIDS patients from this country.
